# Evaluation of a Keratin 1 Targeting Peptide-Doxorubicin Conjugate in a Mouse Model of Triple-Negative Breast Cancer

**DOI:** 10.3390/pharmaceutics13050661

**Published:** 2021-05-05

**Authors:** Azam Saghaeidehkordi, Shiuan Chen, Sun Yang, Kamaljit Kaur

**Affiliations:** 1 Chapman University School of Pharmacy (CUSP), Harry and Diane Rinker Health Science Campus, Chapman University, Irvine, CA 92618-1908, USA; sagha102@mail.chapman.edu (A.S.); syang@chapman.edu (S.Y.); 2 Department of Cancer Biology, Beckman Research Institute of the City of Hope, Duarte, CA 91010, USA; SChen@coh.org

**Keywords:** triple-negative breast cancer, peptide-drug conjugate or peptide-doxorubicin conjugate, keratin 1 targeting, cell-derived xenograft, antitumor efficacy, off-target toxicity

## Abstract

Chemotherapy is the main treatment for triple-negative breast cancer (TNBC), a subtype of breast cancer that is aggressive with a poor prognosis. While chemotherapeutics are potent, these agents lack specificity and are equally toxic to cancer and nonmalignant cells and tissues. Targeted therapies for TNBC treatment could lead to more safe and efficacious drugs. We previously engineered a breast cancer cell targeting peptide **18-4** that specifically binds cell surface receptor keratin 1 (K1) on breast cancer cells. A conjugate of peptide **18-4** and doxorubicin (Dox) containing an acid-sensitive hydrazone linker showed specific toxicity toward TNBC cells. Here, we report the in vivo evaluation of the K1 targeting peptide-Dox conjugate (PDC) in a TNBC cell-derived xenograft mouse model. Mice treated with the conjugate show significantly improved antitumor efficacy and reduced off-target toxicity compared to mice treated with Dox or saline. After six weekly treatments, on day 35, the mice treated with PDC (2.5 mg Dox equivalent/kg) showed significant reduction (1.5 times) in tumor volume compared to mice treated with Dox (2.5 mg/kg). The mice treated with the conjugate showed significantly higher (1.4 times) levels of Dox in tumors and lower (1.3–2.2 times) levels of Dox in other organs compared to mice treated with Dox. Blood collected at 15 min showed 3.6 times higher concentration of the drug (PDC and Dox) in mice injected with PDC compared to the drug (Dox) in mice injected with Dox. The study shows that the K1 targeting PDC is a promising novel modality for treatment of TNBC, with a favorable safety profile, and warrants further investigation of K1 targeting conjugates as TNBC therapeutics.

## 1. Introduction

Triple-negative breast cancer (TNBC) is an important subtype of breast cancer as it is aggressive and has a poor prognosis [1,2]. TNBC is characterized by the absence of estrogen and progesterone receptors and without the overexpression of human epidermal growth factor receptor 2 (HER2). TNBC is a heterogeneous disease with ≈80% of TNBC cells with basal-like gene expression characteristics, such as *KRT5*, *KRT14*, *KRT17,* and *EGFR* [1,3]. Lehmann et al. defined TNBC into four molecular subtypes, namely basal-like 1 (BL1), basal-like 2 (BL2), mesenchymal (M) and, luminal androgen receptor (LAR) [3]. Several potential pathways with deletion (such as *PTEN*) and mutation/amplification (*PIK3CA, KRAS, BRAF, EGFR,* and *MET*) of genes have been identified as druggable molecular alterations in TNBC, but these have not proven to be clinically successful yet [1]. Some success has been seen with the recent approval of poly-ADP ribose-polymerase (PARP) and immune directed checkpoint inhibitors, and an antibody-drug conjugate, sacituzumab govitecan [4].

Chemotherapy remains the primary treatment for TNBC [5]. Anthracyclines alone or sequential treatment with anthracycline, cyclophosphamide, and taxane are used to achieve pathological complete response (pCR) in patients. The benefit of dose-dense (weekly or once every 2 weeks) administration of chemotherapeutics to achieve the highest pCR rates is also emphasized [6]. However, chemotherapy’s effectiveness is limited due to two reasons: (i) intolerable toxicities that emerge after intensive therapy as a result of nonspecific action of the chemotherapeutics in healthy tissues, and (ii) the inherent or acquired resistance of tumor cells to chemotherapeutics that leads to selection and proliferation of resistant cells after first-line treatment. Overall, this leads to poor quality of life in patients and forces the clinicians to use suboptimal doses of the drug to prevent both acute and chronic toxicities. In addition, the resistance of tumor cells to chemotherapeutics increases the chance of survival of migrated tumor cells and progression to fatal metastasis [7].

Different strategies are being developed to target chemotherapeutic agents to improve their specific uptake by cancerous cells in tumors, such as the use of targeting ligands to target specific receptors on cancer cells [8,9,10]. Active targeting of the drug with targeting ligands, like engineered antibodies or tumor homing peptides, allows a higher concentration of the drug to reach the tumor site, thereby increasing efficacy and reducing toxic side effects of the drug [8,9,10]. A drug conjugated to an antibody or a peptide via a linker yields an antibody-drug conjugate (ADC) or a peptide-drug conjugate (PDC). ADC and PDC are new modalities that show promise in the treatment of various cancers [8,9,11]. In recent years, several ADCs have been FDA approved to treat hematologic and solid cancers [9,12]. ADCs and PDCs in the clinical and preclinical development lay promise for the future of this rapidly growing class of drugs in oncology. ADC sacituzumab govitecan was FDA approved for the treatment of metastatic TNBC, where the antibody targets trophoblast cell-surface antigen 2 (Trop-2) present on TNBC cells [12,13,14]. Erythropoietin-producing hepatocellular receptor A2 (EphA2) is a protein from the receptor tyrosine kinase family that serves as a known target for cancer treatment due to its overexpression in tumors and low expression in healthy tissues [15,16]. EphA2 is being utilized for the development of targeted therapies such as ADCs and PDCs [8,17,18,19]. Similarly, several other receptors, such as gonadotropin-releasing hormone (GnRH), LDL receptor-related protein 1 (LRP1), somatostatin subtype-2, and transferrin, are being targeted for delivery of chemotherapeutics to the cancer site [8]. A PDC (ANG1005) of paclitaxel with an LRP1 targeting peptide angiopep-2 is currently undergoing several clinical trials for the treatment of patients with brain metastases from breast cancer [20,21,22]. 

**Figure 1:**
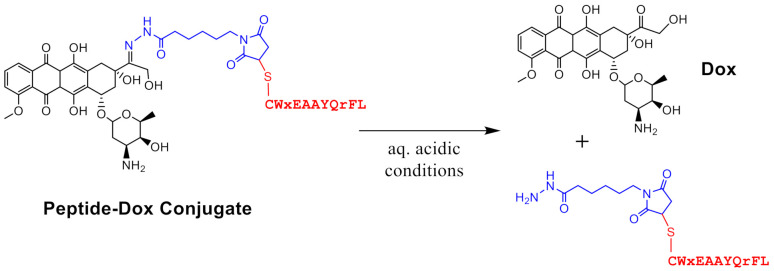
Structure of keratin 1 targeting peptide-Dox conjugate (PDC). The conjugate contains an *N*-acyl hydrazone linker which hydrolyzes under aqueous acidic conditions to give unmodified doxorubicin (Dox) and hydrazide linker with the peptide. Peptide, linker, and Dox are shown in red, blue, and black, respectively.

Keratin 1 (K1) is a novel receptor, present on the surface of cancer cells (breast and neuroblastoma) [23,24,25] and cells that have undergone oxidative stress [26], that is being used for targeted drug delivery. We showed that K1 is present on the surface of MCF-7 breast cancer cells, and a comparison of the total K1 levels in cell lysates using Western blot showed that cancer cells (MCF-7 and MDA-MB-435) have a much higher expression of K1 compared to non-cancerous breast tissue derived epithelial (MCF-10A) cells [25]. We engineered peptides, such as linear **18-4** and cyclic analogues, for specific uptake by breast cancer cells (MCF-7 and MDA-MB-231) via cell surface K1 mediated endocytosis [25,27]. Further, K1 targeting linear peptide **18-4** was used to synthesize four peptide-doxorubicin conjugates with different linker chemistries, such as ester, amide, succinimidyl thioether, and hydrazone [28,29]. We showed specific uptake of the targeted PDCs via receptor mediated endocytosis in MCF-7 and MDA-MB-435-MDR cancer cells [28]. The PDCs with K1 targeting peptide **18-4** were more cytotoxic to TNBC cells (IC_50_ 1.2–4.7 µM) compared to non-cancerous human mammary epithelial MCF-10A cells (IC_50_ 15.1–38.6 µM), while free drug (doxorubicin) was equally cytotoxic to both cancer and non-cancerous cells (IC_50_ 0.24–1.5 µM) [29]. To explore the in vivo efficacy and evaluate the potential of K1 targeting PDC for TNBC treatment, we report here the antitumor activity of one of these peptide-doxorubicin conjugates (Figure 1), where the peptide (**18-4**) is conjugated to Dox via an acid-sensitive N-acyl hydrazone linker in a mouse model for TNBC. TNBC MDA-MB-231 cells were subcutaneously injected into female NOD/SCID mice to generate TNBC cell-derived xenograft models. Mice treated with the conjugate showed better efficacy, pharmacokinetics, and safety profile compared to the Dox treated mice, supporting the future clinical development of K1 targeted PDCs for treatment of TNBC.

## 2. Materials and Methods

### 2.1. Conjugate Solution for Intravenous Injections

Peptide-Dox conjugate (PDC, Figure 1) was synthesized and purified, as described previously [29]. Doxorubicin hydrochloride salt (Dox.HCl) was purchased from LC Laboratories (Woburn, MA, USA), while daunorubicin hydrochloride (Dau) and aldoxorubicin (Aldox) were from MedChem Express (Monmouth Junction, NJ, USA). An analytical reversed-phase high performance liquid chromatography (RP-HPLC) column (4.6 mm × 250 mm, 5 µm) running with a gradient on a Shimadzu LCMS 2020 (Shimadzu Corporation, Columbia, MD, USA) was used for characterization of PDC. The mass spectrum showed two m/z peaks, 717.5 (3+) and 1075.2 (2+), for the conjugate that allowed found mass to be 2149.5 (calculated mass 2148.7) [30]. The UV/Vis spectra for all compounds (≈200 µM) were obtained on UV/Vis spectrophotometer UV-2600/2700 (Shimadzu Corporation, MD, USA). Stock solutions of Dox and conjugate were prepared in 0.9% sterile saline (MWI Animal Health, Boise, ID, USA) and concentration was measured using QuickDrop (Molecular Devices, San Jose, CA, USA) at 481 nm and 495 nm, respectively. The stock solution was diluted to the appropriate concentration (2.5 mg Dox or Dox equivalent/kg) in 0.9% saline solution before injecting. Solutions were prepared fresh or a few days before each injection and kept at −20 °C freezer until use. All solutions were warmed (30–32 °C) prior to injection, and each mouse was intravenously injected with 200 µL solution via tail vein.

### 2.2. Drug Circulation Time

Nonobese diabetic—severe combined immunodeficiency (NOD-SCID) female mice (8-week old) were purchased from Charles River Laboratories (Wilmington, MA, USA). All animal experiments were performed according to the Chapman University Institutional Animal Care and Use Committee (protocol # 1617A008, approval period 16 May 2017–15 May 2020 and protocol # 2020-1148, approval period 20 May 2020–20 May 2023) and in accordance with NIH guidelines. To measure the circulating levels of the drug, after intravenous administration of PDC (2.5 mg Dox equivalent/kg) via tail vein, mice (*n* = 3) were euthanized using metered CO_2_ gas chamber at times 0.25, 2, 4, and 24 h. The blood samples were collected via cardiac puncture and kept at room temperature for 30 min until the blood coagulated [31]. Serum samples were then collected by centrifuging at 3000× *g* for 15 min at room temperature. With the size of mice that were used for the study, cardiac puncture collected at least 250 µL of whole blood, which approximately yielded 100 µL of serum. The conjugate and its metabolite were extracted from serum following the previous method with some modifications [32,33]. Briefly, the serum was diluted with MQ water (300 µL) and mixed with acetonitrile (300 µL). Daunorubicin (20 µL and 150 µM) was added as an internal standard to each sample before organic extraction. The resulting mixture was vortexed for 20 s. It was then centrifuged at 2500× *g* for 3 min. The supernatant was transferred to a new tube containing 1 mL of MQ water. Next, chloroform and isopropanol mixture (4 mL, 1:1) was added, followed by vortex-mixing for 60 s. After centrifugation for 3 min at 2500× *g*, the organic lower layer needed to remain in the tube, and the top layer was carefully removed and discarded. The content in the tube was then dried using SpeedVac Concentrator (Thermo Scientific SPD1010, Waltham, IL, USA), followed by reconstitution in methanol (120 µL). An aliquot (40 µL) was injected into an analytical RP-HPLC column and analyzed using LC/MS. A gradient method was used to elute and characterize the conjugate and its metabolites.

### 2.3. Cell Culture and Tumor Inoculation

Human TNBC MDA-MB-231 cells were purchased from the American Type Culture Collection (ATCC, Manassas, VA, USA). Cells were cultured in DMEM/F12 media (Gibco, New York, NY, USA), supplemented with 10% FBS (Corning) and 1% penicillin/streptomycin (Gibco, NY, USA). Cells were kept in a humidified atmosphere in a 5% CO_2_ incubator maintained at 37 °C. Twenty-one NOD-SCID female mice were used for tumor inoculation. Each mouse (8-week old) was inoculated with 2 million MDA-MB-231 cells in DMEM-F12 media/Matrigel (1:1 ratio, 100 µL) (Corning, Glendale, AZ, USA) by subcutaneous injection into the right flank using 1 mL conjugate syringes and 25 G needles to prevent destruction of cells [32]. The needle was slightly twisted outwards to avoid the content from leaking out. Tumor size was measured twice weekly with a digital caliper in two dimensions (length: largest diameter, width: smaller diameter) and tumor volume was calculated using the formula: ¾ π × L × W^2^ × 1/8 (mm^3^) (L = length; W = width; mm) [34]. 

### 2.4. Drug Treatment

After inoculation, when the volumes of MDA-MB-231 tumor xenografts reached around 100–150 mm^3^, mice were randomly divided into three groups for the experimental treatment. The general health status of each mouse was monitored daily for any signs of distress, such as lethargy, ruffled coat, and ataxia. Body weight was also recorded weekly to assess systemic toxicity associated with the treatments. The mice were subjected to euthanasia if (1) the mouse’s body weight dropped below 15% of its initial weight, (2) the mouse’s tumor became >1.5 cm across in any dimension, or (3) the mouse became lethargic, sick, or unable to feed. Twenty-one mice divided into three groups (*n* = 7) were treated with (i) 0.9% saline solution containing 3% DMSO (treatment group 1), (ii) free Dox (2.5 mg/kg body weight) (treatment group 2), or (iii) peptide-Dox conjugate (2.5 mg DOX equivalent/kg) (treatment group 3). All treatments in 0.9% saline solution containing 3% DMSO were administered intravenously via tail vein (200 µL) every 7th day for a total of six doses. Tumor size was measured twice weekly on the day of injection and three days later. Saline treated mice were euthanized earlier than the DOX or conjugate treated mice (day 32 instead of day 36) due to the large tumor size (volume ≈800 mm^3^) and difficulty in moving and feeding, which is compliant with the euthanasia criteria.

### 2.5. Biodistribution of Drug

For the biodistribution analysis of PDC, mice were euthanized 24 h after the last dose. The tumor xenografts and other tissues/organs (liver, spleen, kidneys, lungs, and heart) were collected from each mouse, and stored at −80 °C for Dox and/or metabolite analysis using LC/MS. Tumors were weighed individually to obtain the average tumor weight for each treatment group. For extraction of Dox and its metabolites from the tissue samples, the samples were thawed, and 100 mg of tissue was homogenized in MQ water (300 µL) using a tissue grinder (BTLab Systems, Saint Louis, MO, USA) [32,33]. The Dox and its metabolites were extracted from the tissue samples as described above for the serum samples. The extracted sample was reconstituted in methanol (120 µL) and centrifuged to collect the supernatant. This was done to remove any insoluble particulates and prevent clogging the RP-HPLC column. The supernatant (40 µL) was injected into an analytical RP-HPLC column and analyzed using Shimadzu LCMS 2020 presented as µg/g tissue.

### 2.6. Statistical Analysis

Data are presented as mean ± standard deviation (SD), and statistical significance of difference was assessed using Student’s *t*-test and one-way analysis of variance (ANOVA) test. The difference was considered statistically significant with *p* < 0.05 and the reported *p* values were from two-tailed tests. All data that required non-linear regression analysis were processed using Microsoft Office Excel or GraphPad Prism, version 9.0.0 (GraphPad software, San Diego, CA, USA).

## 3. Results

### 3.1. In Vivo Stability of Peptide-Dox Conjugate

A gradient method was developed to observe and characterize the PDC (Figure 1) and its metabolites using the LC/MS. An analytical RP-HPLC column running with a 15–55% acetonitrile/water gradient (with 0.05% formic acid) over 41.5 min at a flow rate of 0.4 mL/min was used. As shown in Figure S1 (Supplementary Materials), this method eluted the PDC as three overlapping peaks on the analytical column between 32.8–36.0 min. The eluting peaks were monitored using UV-vis at 481 nm and electrospray mass spectra. The mol. wt. for each of the peaks calculated from the electrospray mass spectra was found to be 2149.5 (calculated 2148.7). The three peaks with the same molecular weight likely represent three solution conformations of the conjugate. The UV-Vis spectrum for the conjugate solution (≈200 µM) was compared to the spectra for Aldox and Dox. It was found that the conjugate displayed similar broad maxima as observed for Aldox and Dox around 480–495 nm (Figure S2).

**Figure 2:**
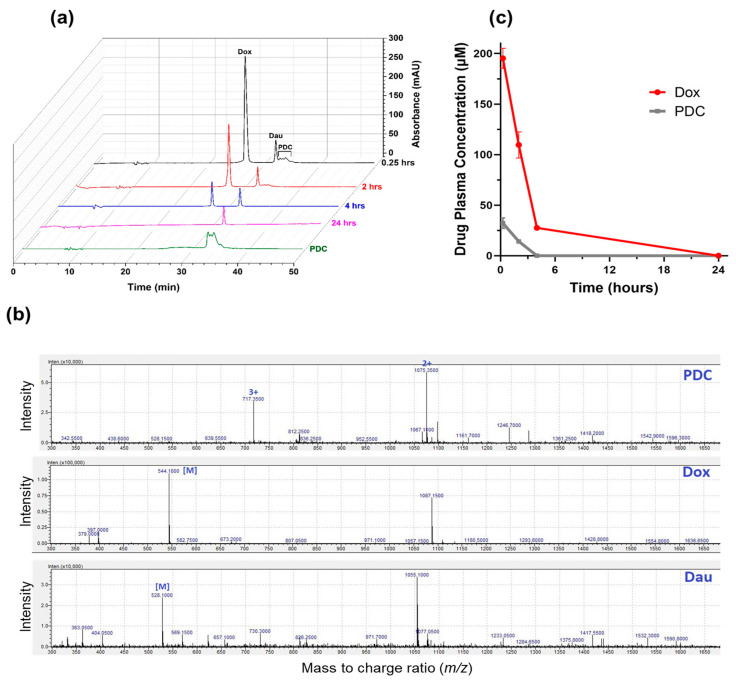
Concentration of total drug (PDC and Dox) after administration of PDC in female (8-week-old) nonobese diabetic—severe combined immunodeficiency (NOD-SCID) mice. Mice (*n* = 3) were administrated PDC (2.5 mg/kg Dox equivalent or 0.088 ± 0.008 µmoles) via tail vein injection, and blood was collected via cardiac puncture at 0.25, 2, 4, and 24 h. The serum (100 µL) from blood was collected. Serum was subjected to organic extraction, and the extract was injected in LC/MS system to detect and quantify PDC and released Dox. No other metabolites of Dox were observed. (**a**) Representative chromatograms for samples after extraction, monitored at λ = 481 nm. Dau was used as an internal standard. Chromatogram of pure PDC (280 µM, 20 µL) is shown for comparison; (**b**) Representative electrospray ionization (ESI) mass spectra of PDC, Dox, and Dau obtained during the LC/MS analysis of one sample out of three at 0.25 min. PDC shows two m/z peaks at 717.35 and 1075.35 with charges +3 and +2, respectively. The found mass (M) from these peaks for PDC was 2149.1 (calcd. 2148.7). For Dox and Dau, the found mass were 544.1 (calcd. 543.5) and 528.1 (calcd. 527.5), respectively; (**c**) Average serum concentration (µM) of PDC and Dox at each time interval obtained from the LC/MS data. Data shown is mean ± SD.

In the design of the PDC, the Dox and peptide were linked via an N-acyl hydrazone bond on one end and a succinimide thioether bond at the other end (Figure 1). While thioether is considered non-cleavable, the acid-sensitive hydrazone was incorporated to facilitate the release of the unmodified drug at the target tumor site, i.e., in the tumor microenvironment or after the cellular uptake in the acidic environment of endosomes or lysosomes [8]. To evaluate the circulation time of the drug after systemic administration of PDC, the PDC (2.5 mg Dox equivalent/kg) was injected intravenously via the tail vein in mice. Blood samples were collected via cardiac puncture at different time intervals. PDC and its metabolites were extracted following a series of steps and analyzed using LC/MS (Figure 2). At 15 min after PDC injection, the PDC and Dox were detected in blood serum. At 2 h, both were still present; however, the concentration for each dropped substantially (2.3× less PDC and 1.8× less Dox) compared to at 15 min (Figure 2a,c). At 4 h after injection, only Dox was observed. By 24 h after injection, Dox diminished to an undetectable level in the blood. Based on the total concentration of the drug (PDC and Dox) present in the blood, the circulation half-life of the drug (PDC and Dox) was estimated as ≈1.7 h after PDC administration. This is substantially longer than Dox’s half-life, which was eliminated from the blood within minutes after Dox injection [35]. We observed that mice injected with PDC had 3.6 times more drug (227.5 ± 15 µM) in blood compared to drug levels (63.4 ± 4.2 µM) in mice injected with free Dox at 15 min (Table S1 and Figure S3). The results demonstrate higher concentration and a longer circulation time of the drug (PDC and Dox) after PDC administration (2.5 mg Dox equivalent/kg) compared to the drug (Dox) after Dox administration (2.5 mg Dox/kg) to mice. Here, the total drug concentration (PDC and Dox) in the blood was determined after PDC administration, as the experiment does not allow estimation of PDC alone in blood. This is because PDC was likely hydrolyzed to Dox while blood was being processed by different steps, such as blood coagulation, centrifugation, and extraction. This is also the likely reason for a large Dox peak and a small PDC peak at 15 min (Figure 2a) and does not reflect the true ratio of the two in blood at 15 min.

### 3.2. TNBC Tumor Growth Rate in a Subcutaneous Cell-Derived Xenograft Model

**Figure 3:**
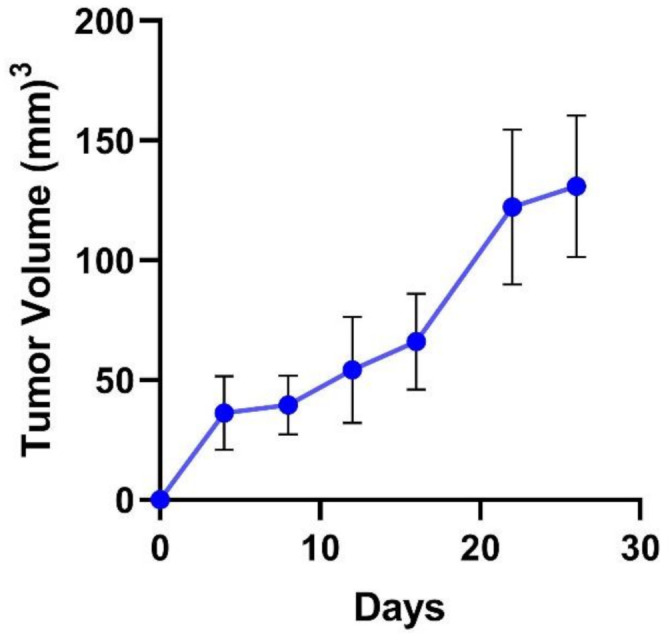
The growth curve of established tumors (*n* = 21, mean ± SD) using direct subcutaneous injection of MDA-MB-231 cells (2 million in media and Matrigel, 100 µL, 1:1) into the right flank of female NOD-SCID mice (8-week-old).

Human breast cancer MDA-MB-231 cells were selected for establishing cell-derived xenograft (CDX) in female NOD-SCID mice. MDA-MB-231 cells are aggressive with high metastatic potential and belong to mesenchymal (M) TNBC subtype (Basal B) [3,36,37]. The gene expression profile for these cells is associated with stem cells and mesenchymal stem cell-specific markers. Breast tumor xenografts were established by subcutaneously injecting two million MDA-MB-231 cells in the right flank of mice. The subcutaneous xenografts, unlike the orthotopic xenografts, do not metastasize, however are still a popular model for studying antitumor efficacy [38]. Tumor growth was monitored by measuring the tumor size twice a week. As shown in Figure 3, the tumor volume reached 130.9 ± 30 mm^3^ in 26 days. A high variation in volume of the xenograft tumors was observed, which is a characteristic of subcutaneous implanted xenografts [39]. On day 4 post-cell injection, tumors were observed by the naked eye growing on top of the mammary gland. The tumor volume increased gradually with a round to oval-shaped bump. Throughout the 26-day tumor growth period, the behavior and general appearance of mice remained normal.

### 3.3. In Vivo Antitumor Effect of Peptide-Dox Conjugate

**Figure 4:**
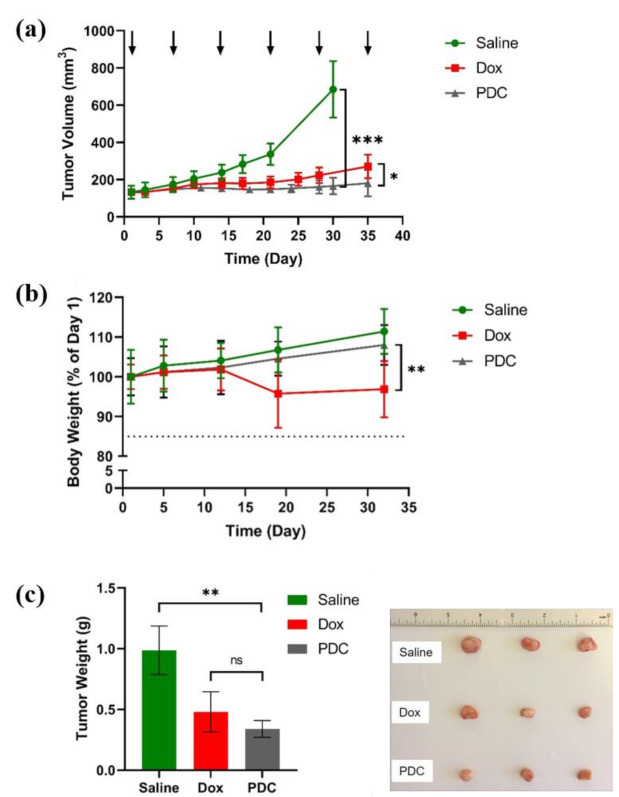
Effects of PDC on tumor growth using a mouse model of TNBC. NOD-SCID female mice bearing xenograft human MDA-MB-231 tumors were treated with PDC (2.5 mg Dox equiv./kg), Dox (2.5 mg/kg), or saline (0.9% normal saline). (**a**) Tumor volumes measured over the duration of treatment. The treatment regimen included six tail-vein injections administered weekly (as indicated by arrows). Results are presented as mean ± SD; *n* = 7; * *p* < 0.05, *** *p* < 0.001, Student *t*-test and one-way ANOVA test (at day 30 or day 35); (**b**) Average body weight of mice for each treatment group. Results are presented as mean ± SD; *n* = 7; ** *p* < 0.01, Student *t*-test and one-way ANOVA test (at day 32); (**c**) average tumor weight after mice were euthanized and tumors collected. Results are presented as mean ± SD; *n* = 7; ** *p* < 0.01, ns non-significant, Student *t*-test and one-way ANOVA test. (RHS of c) representative images of the tumor xenografts collected from three groups.

After the tumor xenografts reached a volume of around 100–150 mm^3^, mice were randomized into three groups (*n* = 7), namely, saline (negative control), free doxorubicin (positive control), and hydrazone PDC. A low dose of 2.5 mg/kg Dox [33,40] or 2.5 mg/kg Dox equivalent for PDC was chosen to study the antitumor efficacy in vivo. Mice were intravenously administered treatment by tail vein every seventh day for six doses. Compared to the saline group, the PDC reduced tumor growth significantly (3.8 times) on day 35 after treatment, whereas the reduction of tumor growth after free Dox treatment was 2.5 times (Figure 4a), suggesting the PDC, at the same equivalent dose, was more potent than the free Dox.

In addition, the mice treated with PDC remained in overall good health condition, as evidenced by the general appearance, behavior, diet consumption, and body weight. On day 32 during the treatment period, there were no significant differences observed between the PDC and saline groups in the average body weight (*p* > 0.05) (Figure 4b). However, the mice treated with Dox showed significant body weight loss (reduced by 11.2%) compared to the PDC group. Twenty-four hours after the last treatment with PDC or free Dox, mice were euthanized. Mice treated with saline were euthanized on day 32 because of the tumor size per IACUC policy and the conditions for euthanasia. Tumor and other major tissues were collected and weighted for further analysis. The mice with PDC treatment exhibited a greater reduction (three times reduction compared to saline) of tumor weight compared to that of free Dox treated (two times reduction compared to saline) (Figure 4c).

### 3.4. In Vivo Tissue Biodistribution Study

**Figure 5:**
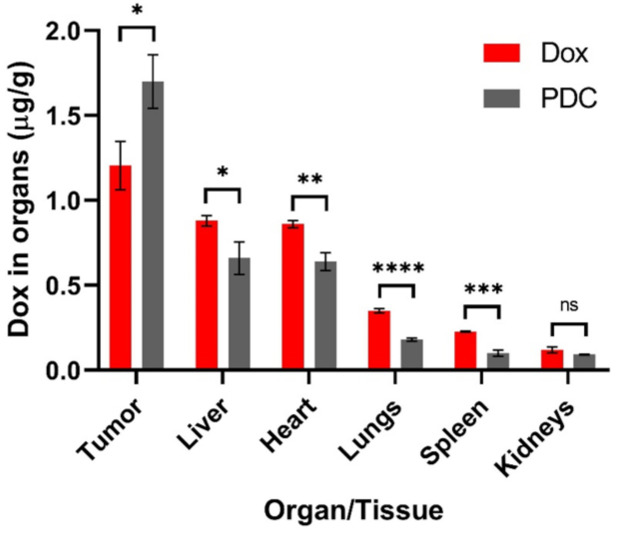
Biodistribution of Dox in tumor and other tissues at 24 h post injection (Dox or PDC). Data are presented as mean ± SD; *n* = 3; * *p* < 0.05, ** *p* < 0.01, *** *p* < 0.001, **** *p* < 0.0001, ns non-significant; Student *t*-test and one-way ANOVA were used. The significance level (α) was set at 0.05.

The tumor and five organ tissues (liver, spleen, kidneys, lungs, and heart) were collected and subjected to organic extraction to isolate and quantify PDC and/or its metabolites using LC/MS. At 24 h after the last treatment, only Dox was detected in all the samples. There were no PDC or other metabolites detected using the LC/MS method. In the PDC treated mice, the Dox content was much higher in tumor samples (1.70 ± 0.16 µg/g) compared to other organs (0.66–0.09 µg/g) (Figure 5).

When comparing the PDC and Dox treatment groups, the Dox concentrations in isolated tumors of the PDC treated mice were significantly increased (1.4-fold more Dox). In contrast, in other tissues, Dox levels were lower in the PDC treated mice compared to the free Dox group, highlighting the targeted delivery of the conjugate to the tumor tissue. Mice treated with the conjugate showed significantly lower levels of Dox in liver (1.3 times), heart (1.4 times), lungs (1.9 times), and spleen (2.2 times) compared to mice treated with free Dox (*p* < 0.05). The levels in kidney were also lower (1.3 times) in PDC treated mice but not statistically significant. Notably, Dox levels were higher in the heart than in the kidney, spleen, and lungs for both the PDC and Dox treated mice. This is likely due to the cardiotoxicity of Dox as it tends to accumulate in the heart [41,42]. Higher levels of Dox in the heart compared to other organs were also observed by others after mice were treated with free Dox [43,44,45]. 

## 4. Discussion

Peptide-drug conjugates represent a novel class of targeted drug delivery modalities in cancer therapy [8,46,47]. In a PDC, the targeting peptide and the linker play an important role in enhancing the efficacy of the drug. We used a linear **18-4** peptide that was engineered to bind breast cancer cells via the cell-surface K1 receptor [23,28] The cellular uptake of peptide **18-4** and its PDC takes place via receptor-mediated endocytosis in breast cancer cells [25,48]. A PDC (Figure 1) using linear **18-4** peptide was synthesized to evaluate the specificity of the conjugate toward TNBC cells [29]. The conjugate showed specific toxicity toward the TNBC cells (IC_50_ 1.2–2.2 µM) and was 9-fold less toxic to normal breast tissue-derived MCF-10A cells (IC_50_ 15.1 µM). In comparison, Dox was equally toxic to both TNBC and normal cells. In this study, we demonstrate the in vivo specificity of the PDC for the tumor tissue. Using CDX mice models, we show significantly improved efficacy of PDC in reducing tumor volume compared to free Dox (Figure 4a). The results here validate the use of peptide **18-4** for targeting TNBC. We recently reported a cyclic analogue of **18-4** (N- to C-terminal cyclized WXEAAYQkFL) that targets TNBC 2–3 fold better than the linear **18-4** peptide [27]. A new PDC with the cyclic peptide could further enhance the efficacy of Dox for TNBC treatment. In addition to targeted drug delivery, peptide **18-4** and its cyclic analogue could be used to deliver drug-carrying micelles, siRNA, imaging agents, or diagnostics to TNBC cells. The use of the cyclic peptide for imaging of cells expressing K1 in intact tumor tissues was recently reported [49]. In addition, we previously reported that peptide **18-4** conjugated liposomes displayed superior antitumor activity compared to non-targeted Dox liposomes [32]. These studies highlight the use of peptide **18-4** and analogues for targeting novel cell surface receptor K1 in TNBC.

The PDC (Figure 1) contains an EMCH (N-ε-maleimidocaproic acid hydrazide) linker which has an acid-sensitive hydrazone and a maleimide group. Hydrazone was selected to facilitate the release of unmodified Dox at the cancer site, while maleimide allowed easy conjugation of the peptide via its cysteine thiol. A derivative of Dox with EMCH, called aldoxorubicin (Aldox), was used to form a protein-drug conjugate with endogenous albumin [50,51]. After systemic administration, the conjugate is formed between Aldox and the circulating albumin in the blood for site-selective delivery of Dox to the tumor. This protein-drug conjugate allowed greater doses (3–4×) of Dox to be administered with minimal cardiotoxicity [47,52]. The only disadvantage of this approach is off-target toxicities due to possible reactions of Aldox with cysteine and lysine of other endogenous proteins. Regardless, Aldox continues to display promising results, including its current advanced phase clinical trials for the soft tissue sarcoma [53,54].

The hydrazone group in the conjugate facilitates the release of the drug under mildly acidic conditions; however, it also makes it less stable. Previously, we found that when incubated with 25% human serum, the conjugate showed a half-life of ≈6 h, mainly cleaving at the hydrazone bond, giving unmodified Dox and the peptide with the linker portion (Figure 1) [29]. After intravenous administration in mice, the PDC showed a half-life of around 1.7 h in blood (Figure 2c), which incidentally is much longer than the half-life of Dox (in min) [35]. The PDC led to an increase in the circulation time of the drug. Additionally, the amount of Dox in tumor tissue in PDC treated mice was significantly higher than the Dox treated mice (Figure 5). These results show higher specificity of PDC for the tumor tissue with less systemic toxicities compared to free Dox (Figure 4b). The usage of Dox is greatly limited by its cardiotoxic effects, ranging from occult changes in myocardial structure to severe cardiomyopathy and heart failure requiring cardiac transplantation [41]. Of note, the Dox levels in hearts after PDC treatment were much lower (1.4 times) compared to free Dox treatment group. The use of PDC may reduce the risk of cardiotoxicity without compromising its anti-tumor activities.

PDCs targeting different overexpressed receptors in cancer cells are being developed. TNBC has been a difficult breast cancer subtype to target mainly due to the heterogeneity of this cancer subtype and lack of clinically validated markers. Recently, an ADC sacituzumab govitecan (targeting cell-surface Trop-2) was approved for the treatment of metastatic TNBC [12,13,14]. Trop-2 is a glycoprotein present in elevated amounts on the surface of cancer cells in solid tumors affecting multiple cancer signaling pathways [13]. PDC targeting other overexpressed receptors such as EphA2 [15,17,19], LRP-1 [20,21], and GnRH [55] are under development for metastatic breast cancer. The PDC (Figure 1) studied here targets cell-surface K1 receptor. Keratins are cytoskeleton proteins; however, the cell-surface presence of keratins is found in several cancer cells, including breast (K1), neuroblastoma (K1), and colon (keratin 8) cancer cells [23,24,25,56], and these proteins are linked to tumor metastases. The promising results with the PDC warrant further investigation of K1 targeting conjugates as a new modality for targeted TNBC treatment.

## 5. Conclusions

We show that the PDC with K1 targeting peptide and acid-sensitive hydrazone linker is more efficacious and less systemically toxic compared to free Dox in mice with TNBC xenografts. The peptide and linker chemistry used here can be applied to prepare new PDCs with different chemotherapeutic agents (such as paclitaxel and exatecan) as potential therapeutics for TNBC. The PDC increases the circulation time for the drug in blood compared to free Dox, along with increased concentration of the drug in tumor. The specificity and lower systemic toxicity of the PDC were demonstrated by the lower levels of Dox in non-tumor tissues (such as liver, heart, lung, and spleen) in comparison to free Dox. The study demonstrates the feasibility of achieving a better therapeutic option for TNBC using PDC that target a novel receptor K1.

## Data Availability

Not applicable.

## Supplementary Materials

The following are available online at https://www.mdpi.com/article/10.3390/pharmaceutics13050661/s1, Figure S1. Chromatogram for the peptide-Dox conjugate (PDC) obtained after injection of pure PDC (280 μM, 20 μL) into the LC/MS running with a reversed-phase analytical column (4.6 mm × 250 mm, 5 μm) with an acetonitrile/water (with 0.05% formic acid) gradient as shown; Figure S2. UV-vis spectra for Dox, Aldox, peptide, and PDC at ~200 μM concentration using UV/Vis spectrophotometer UV-2600/2700 (Shimadzu, USA); Figure S3. Chromatogram showing plasma concentration of Dox after administration of Dox in female 8–9 week-old NOD-SCID mice; Table S1. Concentration of PDC and Dox in blood from mice (*n* = 3) after intravenous injection.

## Abbreviations

Aldox	aldoxorubicin
ADC ANOVA	antibody-drug conjugate analysis of variance
CDX	cell-derived xenograft
Dau	daunorubicin
Dox	doxorubicin
EphA2	erythropoietin-producing hepatocellular receptor A2
GnRH	gonadotropin-releasing hormone
K1	keratin 1
LC/MS	liquid chromatography-mass spectrometry
LRP1	LDL receptor-related protein 1
NOD-SCID	nonobese diabetic—severe combined immunodeficiency
PARP	poly-ADP ribose-polymerase
pCR	pathological complete response
PDC	peptide-drug conjugate or peptide-doxorubicin conjugate
RP-HPLC	reversed-phase high performance liquid chromatography
TNBC	triple-negative breast cancer
Trop-2	trophoblast cell-surface antigen 2
