# Supplementary material for: Evaluation of a Keratin 1 Targeting Peptide-Doxorubicin Conjugate in a Mouse Model of Triple-Negative Breast Cancer
Source: Pharmaceutics. 2021 May 5;13(5):661. doi: 10.3390/pharmaceutics13050661 (PMC8148172; doi:10.3390/pharmaceutics13050661)
Supplement: Supplementary file 1 [file pharmaceutics-13-00661-s001.zip › pharmaceutics-1180621-supplementary.pdf]

pharmaceutics

MDPI

Supplementary Materials: Evaluation of a Keratin 1 Targeting Peptide-Doxorubicin Conjugate in a Mouse Model of Triple-Negative Breast Cancer

Azam Saghaeidehkordi, Shiuan Chen, Sun Yang and Kamaljit Kaur

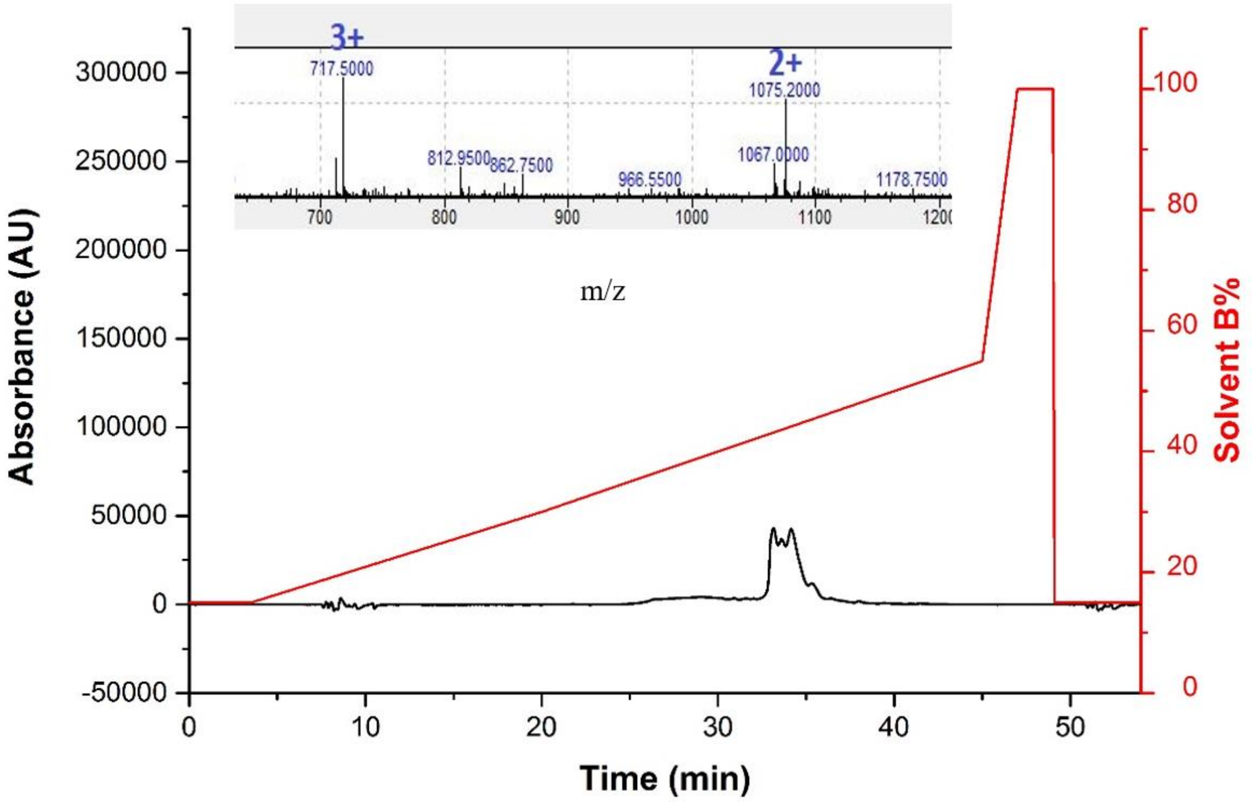

Figure S1. Chromatogram for the peptide-Dox conjugate (PDC) obtained after injection of pure PDC (280 µM, 20 µL) into the LC/MS running with a reversed-phase analytical column (4.6 mm × 250 mm, 5 µm) with an acetonitrile/water (with 0.05% formic acid) gradient as shown. The inset shows the electrospray mass spec of the peak at 33 minutes.

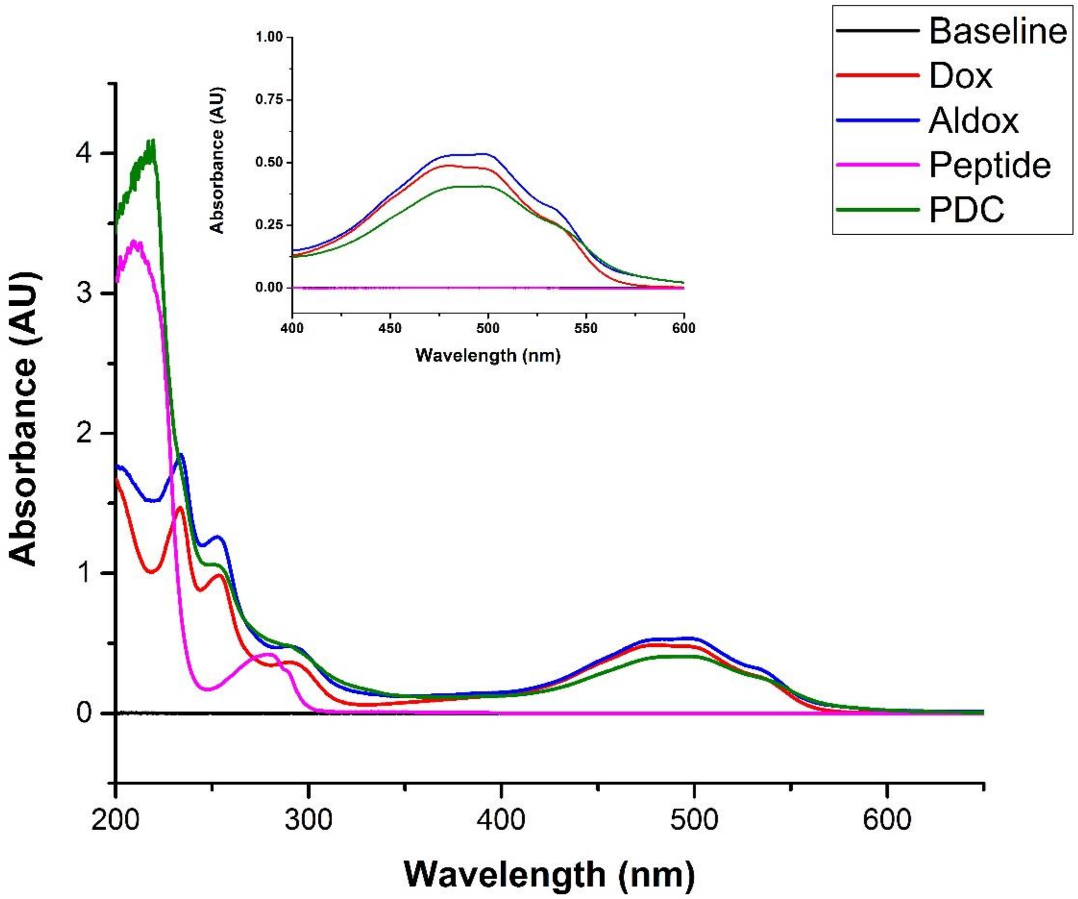

Figure S2. UV-vis spectra for Dox, Aldox, peptide, and PDC at ~200 μM concentration using UV/Vis spectrophotometer UV-2600/2700 (Shimadzu, USA).

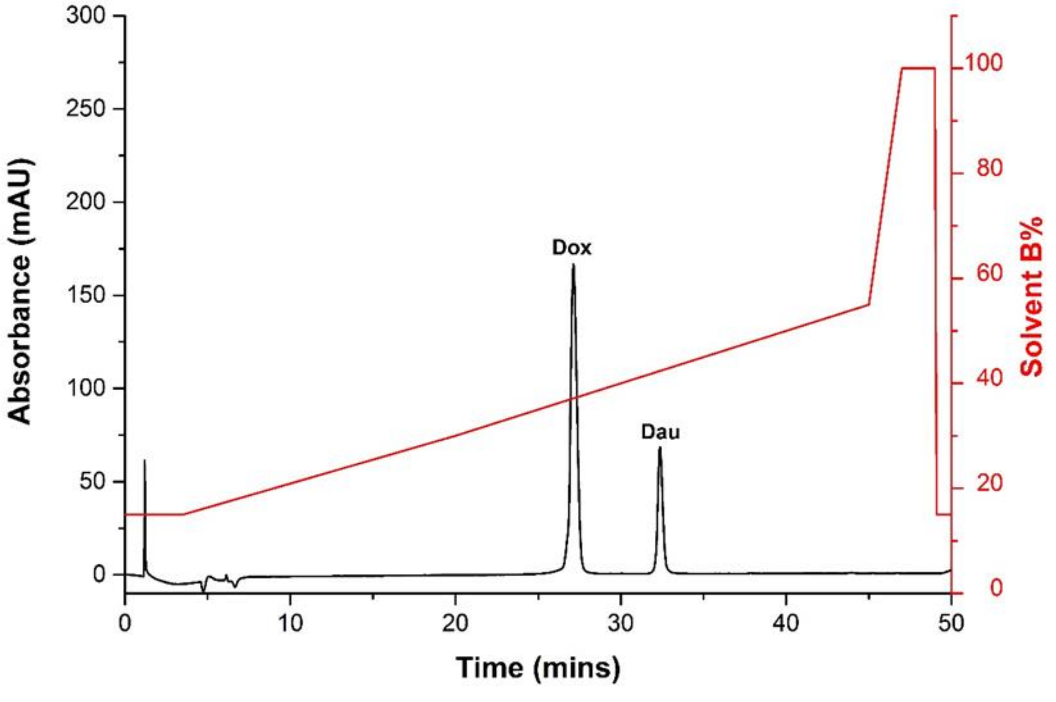

Figure S3. Chromatogram showing plasma concentration of Dox after administration of Dox in female 8–9 week-old NOD-SCID mice. Mice (n = 3) were administrated Dox (2.5 mg/kg Dox) via tail vein injection and blood was collected via cardiac puncture at 0.25 h. The serum (100 μL) from blood was collected. Serum was subjected to organic extraction, and the extract was injected in LC/MS system to detect and quantify Dox No other metabolites of Dox were observed with the LC/MS in the sample. A representative chromatogram monitored at λ = 481 nm for samples from one of the mice is shown,

which shows the presence of Dox in blood at 0.25 h. Electrospray ionization (ESI) mass spectra were used to confirm the identity of Dox and Dau (internal standard) in the LC/MS.

Table S1. Concentration of PDC and Dox in blood from mice (*n* = 3) after intravenous injection.

Drug Injected in Mice (*n* = 3)	Time for Blood Draw after i.v. Injection (h)	Concentration of the Total Drug Detected in Blood * (μM)
		**Total Drug = ([PDC] + [Dox])**
PDC (2.5 mg Dox equiv./kg)	0.25	227.5 ± 15.0 (32.2 ± 5.2 + 195.2 ± 9.9)
PDC (2.5 mg Dox equiv./kg)	2	123.8 ± 14.3 (14.2 ± 1.3 + 109.6 ± 13.0)
PDC (2.5 mg Dox equiv./kg)	4	27.6 ± 2.2 (0 + 27.6 ± 2.2)
PDC (2.5 mg Dox equiv./kg)	24	0
		**Total Drug = [Dox]**
Dox (2.5 mg Dox/kg)	0.25	63.4 ± 4.2

* Calculated using the area under the curve (AUC) for the peaks observed in HPLC chromatograms (Figure 2a and S3). The PDC and Dox concentrations were obtained relative to the known concentration of internal standard daunorubicin.
